# Metastable β-Bi_2_O_3_ Nanoparticles with Potential for Photocatalytic Water Purification Using Visible Light Irradiation

**DOI:** 10.1002/open.201300013

**Published:** 2013-07-02

**Authors:** Maik Schlesinger, Marcus Weber, Steffen Schulze, Michael Hietschold, Michael Mehring

**Affiliations:** [a] Fakultät für Naturwissenschaften, Institut für Chemie, Professur Koordinationschemie, Technische Universität Chemnitz, Straße der Nationen 6209111 Chemnitz (Germany) E-mail: michael.mehring@chemie.tu-chemnitz.de; [b] Fakultät für Naturwissenschaften, Institut für Physik, Professur Analytik an Festkörperoberflächen, Technische Universität Chemnitz, Reichenhainer Straße 7009126 Chemnitz (Germany)

**Keywords:** bismuth oxide nanoparticles, bismuth oxido clusters, organic pollutants, photocatalysis, visible light irradiation

## Abstract

Photocatalytic studies under visible light irradiation using nanosized β-Bi_2_O_3_ are reported. β-Bi_2_O_3_ nanoparticles are prepared starting from the well-defined bismuth oxido cluster [Bi_38_O_45_(OMc)_24_(DMSO)_9_]⋅2 DMSO⋅7 H_2_O (OMc=O_2_CC_3_H_5_) using a straightforward hydrolysis and annealing protocol. Powder X-ray diffraction studies, transmission electron microscopy, diffuse reflectance UV/Vis spectroscopy and nitrogen adsorption measurements (using the Brunauer–Emmett–Teller (BET) theory) are used for the characterization of the as-prepared β-Bi_2_O_3_. By time-dependent annealing, the crystallite size can be controlled between (17±2) nm and (45±5) nm with BET surface areas of 7 to 29 m^2^ g^−1^. The indirect band gap of the as-prepared β-Bi_2_O_3_ amounts to (2.15±0.05) eV. The decomposition rates for rhodamine B (RhB) solutions are in the range of 2.46×10^−5^ to 4.01×10^−4^ s^−1^ and depend on the crystallite size, amount of catalyst and concentration of RhB. Photocorrosion experiments have shown the formation of Bi_2_O_2_CO_3_ after several catalytic cycles. However, the catalyst can be recycled to phase-pure β-Bi_2_O_3_ nanoparticles by annealing for one hour under argon atmosphere at 380 °C. Furthermore, the photocatalytic activity of as-prepared β-Bi_2_O_3_ nanoparticles for the decomposition of phenol, 4-chlorophenol, 2,4-dichlorphenol, 4-nitrophenol, triclosan and ethinyl estradiol is demonstrated.

## Introduction

Since the discovery of the Honda–Fujishima effect in 1972,[1] the research in the field of semiconductor photocatalysis has evolved into two disciplines, the photolysis of water to obtain hydrogen and oxygen from water[2] and the photocatalytic oxidation of pollutants.[3] Notably, photocatalytic degradation of organic dyes and pollutants might become one of the main aspects in modern decentralized purification systems for air and water. Until now, TiO_2_ seems to be the most promising material for such purification systems based on its environmentally benign nature, commercial availability and photochemical stability. However, as a result of the band gap of 3.0–3.2 eV only approximately 7 % of the sunlight (*λ*≤380 nm) can be effectively used. Several approaches have been reported to improve the photocatalytic activity and include the addition of precious metals such as platinum, gold or silver,[4] which makes an industrial process quite expensive (see ref. [5]). Thus, in terms of a sustainable “green chemistry” approach, it is worth to study other nontoxic semiconductor materials which show better response in the visible light region without addition of noble metals. Bismuth-based materials, such as BiVO_4_,[6] BiOCl,[7] Bi_2_O_2_CO_3_,[8] Bi_2_MoO_6_,[9] Bi_2_WO_6_[6c, 10] and Bi_2_Sn_2_O_7_,[11] were previously reported to show promising photocatalytic activities under visible light. Notably, pure bismuth oxide, namely α-Bi_2_O_3_[6c, 12] and the metastable polymorphs β-Bi_2_O_3_[12e, 13] and δ-Bi_2_O_3_,[14] exhibit photocatalytic behavior as well, and their large scale production seems to be interesting with regard to the commercial availability of bismuth (see ref. [5]). Among the bismuth oxide polymorphs, β-Bi_2_O_3_ is the most active heterogeneous photocatalyst. However, the controlled synthesis of monodisperse β-Bi_2_O_3_ nanoparticles is still a challenge. We have recently reported a strategy that is based on a straightforward hydrolysis route starting from well-defined, nanoscaled bismuth oxido clusters.[13e] The structural relationship between the bismuth oxido clusters and β-Bi_2_O_3_ is the key point for the rather mild synthesis method (see Figure S1), which is based on fast hydrolysis at room temperature followed by short time annealing at elevated temperature.[15] Note, that starting from easily accessible [Bi_38_O_45_(OMc)_24_(DMSO)_9_]⋅2 DMSO⋅7 H_2_O[16] (OMc=O_2_CC_3_H_5_), the synthesis of β-Bi_2_O_3_ nanoparticles with high yield on a multigram scale is possible, and preliminary investigations have shown that β-Bi_2_O_3_ nanoparticles prepared by this approach show promising activity in the photocatalytic degradation of organic dyes in aqueous solution.[13e]

**Scheme 1:**
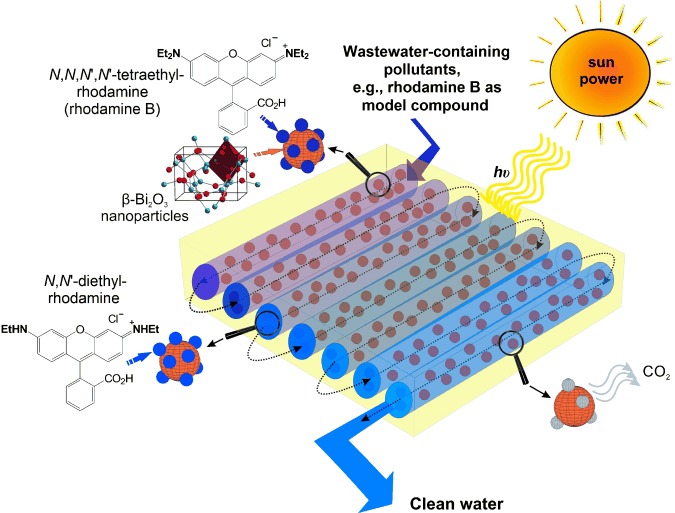
Illustration of a potential tube reactor for water purification using sun light irradiation and β-Bi_2_O_3_ nanoparticles as photocatalysts. Similar systems were already tested in pilot-plant scale, e.g., HIDROCEN (Madrid, Spain).[3f, 19]

Herein, we report detailed studies on the photocatalytic behavior of the as-prepared β-Bi_2_O_3_ nanoparticles under visible light irradiation (Scheme 1). The influence of the crystallite size, the catalyst and dye concentration is investigated by using rhodamine B (RhB) as model pollutant.[17] Additionally, the activity of β-Bi_2_O_3_ is tested using typical organic pollutants, such as phenol, 4-chlorophenol, 2,4-dichlorophenol, 4-nitrophenol, triclosan and ethinyl estradiol, in water.[18]

## Results and Discussion

### Influence of particle size

**Figure 1:**
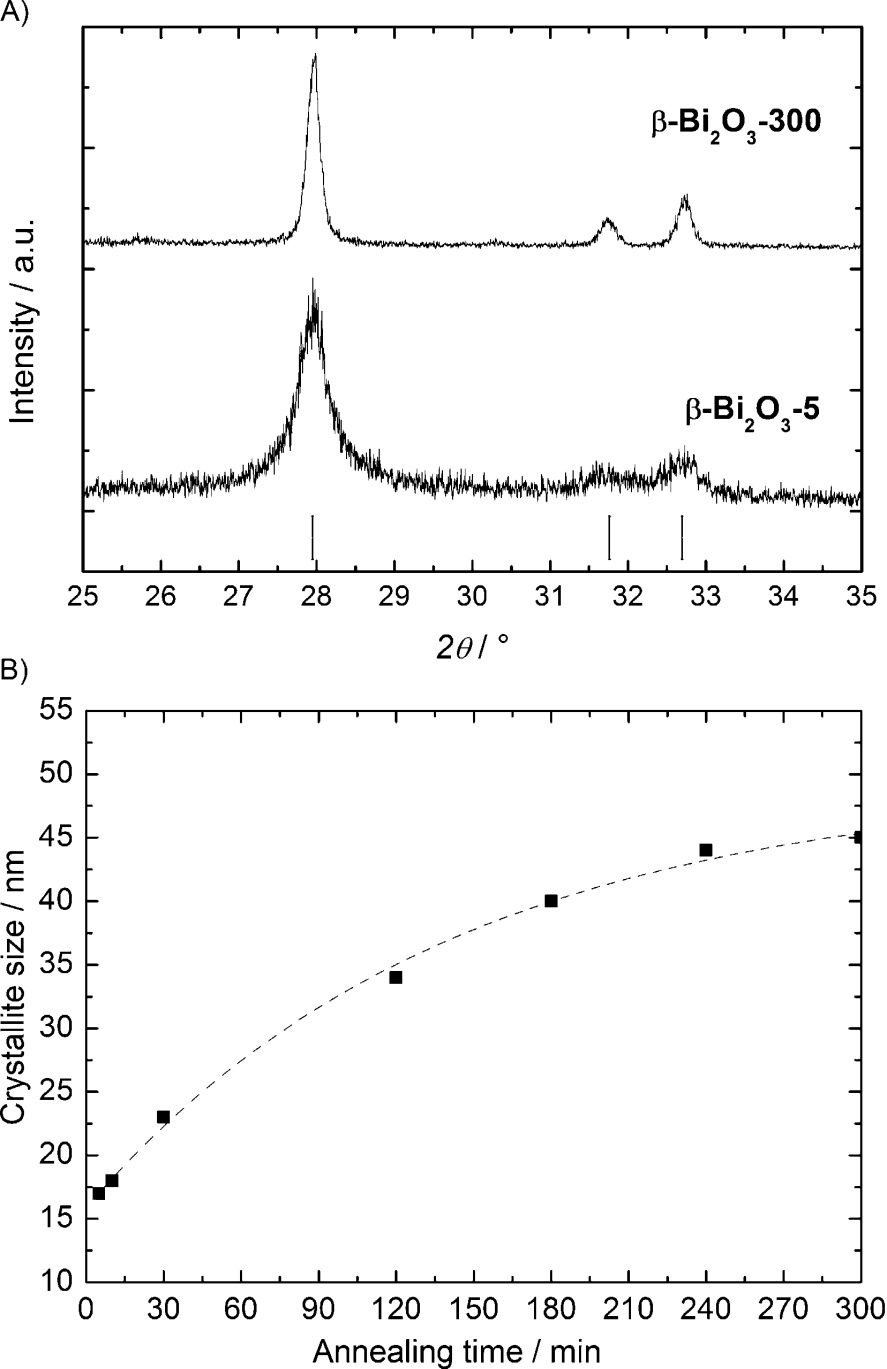
A) Cutout of PXRD patterns of **β-Bi_2_O_3_-5** and **β-Bi_2_O_3_-300** to show the broadening of the reflexes in dependence of the annealing time at 370 °C (reference: β-Bi_2_O_3_, ICDD 00-027-0050). B) A plot of the particle size determined by the Scherrer equation versus annealing time at 370 °C.

**Figure 2:**
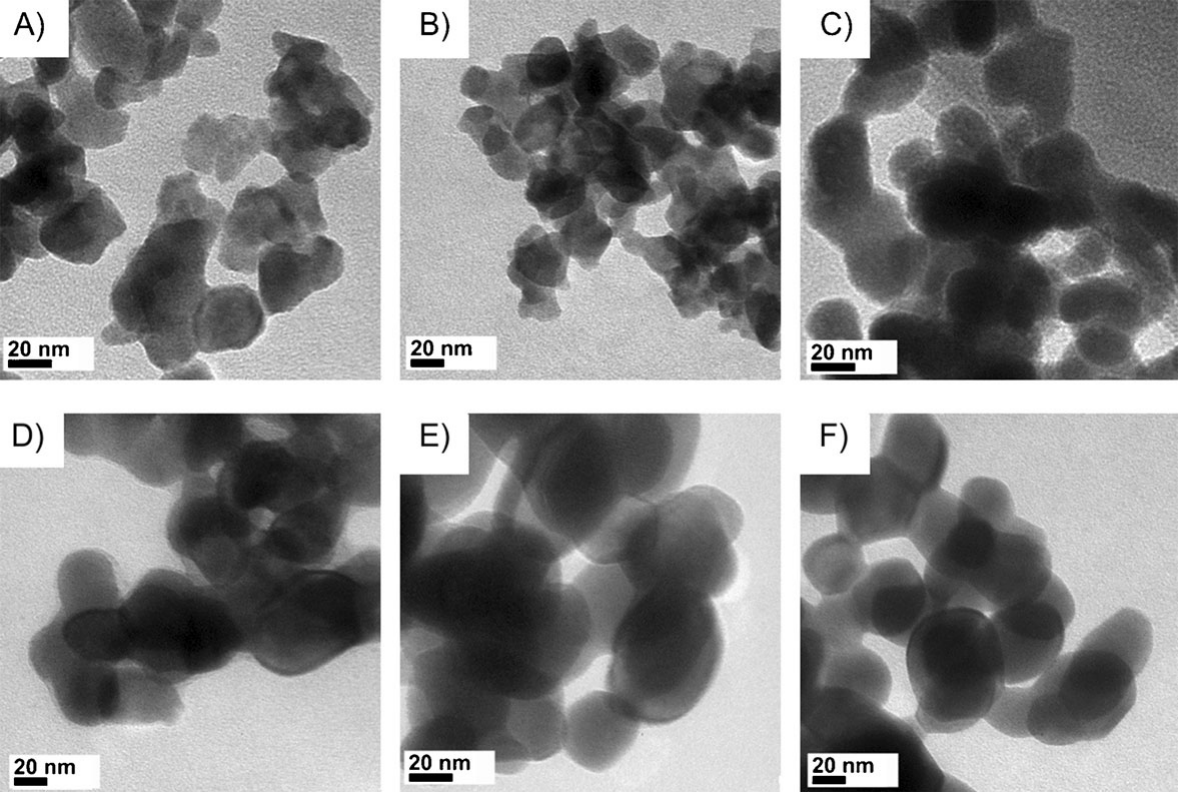
TEM images of the samples A) **β-Bi_2_O_3_-5**, B) **β-Bi_2_O_3_-10**, C) **β-Bi_2_O_3_-30**, D) **β-Bi_2_O_3_-120**, E) **β-Bi_2_O_3_-180** and F) **β-Bi_2_O_3_-300**.

The hydrolysis of [Bi_38_O_45_(OMc)_24_(DMSO)_9_]⋅2 DMSO⋅7 H_2_O with an aqueous sodium hydroxide solution results in the formation of an amorphous powder, which is annealed at 370 °C in an argon atmosphere to give pure β-Bi_2_O_3_ (Figure 1, see Figures S2–S8 in the Supporting Information). The formation of pure β-Bi_2_O_3_ was additionally checked by electron diffraction measurements of samples **β-Bi_2_O_3_-5** (see Figure S9) and **β-Bi_2_O_3_-300**. The composition of Bi_2_O_3_ was confirmed by electron dispersive X-ray spectroscopy (Bi 89.5 wt %; O 10.5 wt %). By increasing the annealing time at the same temperature from five to 300 minutes, the crystallite size of the as-prepared β-Bi_2_O_3_ nanoparticles increased moderately. Values between 17±2 nm (**β-Bi_2_O_3_-5**) and 45±5 nm (**β-Bi_2_O_3_-300**) were obtained. A plot of crystallite size versus annealing time results in a curve which can be described by a function of the type “*a*⋅(1−*b*⋅e^−*κt*^)” (Figure 1). Transmission electron microscopy (TEM) images of the as-prepared β-Bi_2_O_3_ show partially agglomerated particles with particle sizes of (20±3) nm for **β-Bi_2_O_3_-5**, (21±3) nm for **β-Bi_2_O_3_-10**, (23±4) nm for **β-Bi_2_O_3_-30**, (33±4) nm for **β-Bi_2_O_3_-120**, (38±6) nm for **β-Bi_2_O_3_-180** and (40±7) nm for **β-Bi_2_O_3_-300** (Figure 2), which is in line with the results obtained from powder X-ray diffraction (PXRD).

**Figure 3:**
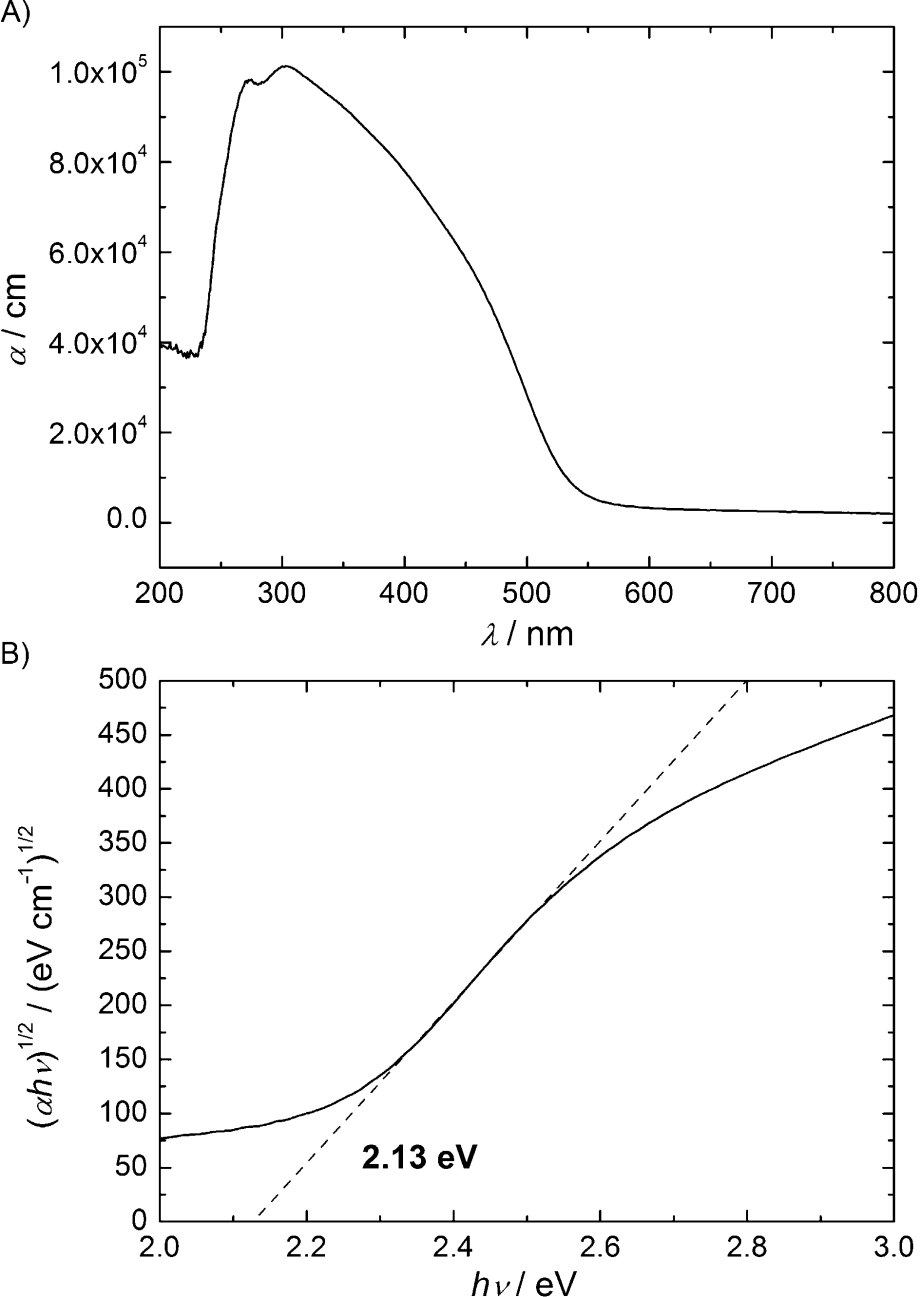
A) Plot of the absorption coefficient versus wavelength and B) (*αhυ*)^1/2^ versus *hυ* for **β-Bi_2_O_3_-10** indicating an indirect band gap semiconductor behavior.

The decrease of the crystallite size from (45±5) nm for **β-Bi_2_O_3_-300** to (17±2) nm for **β-Bi_2_O_3_-5** results in an increase of the Brunauer–Emmett–Teller (BET) surface areas from 7 m^2^g^−1^ (**β-Bi_2_O_3_-300**) to 29 m^2^g^−1^ (**β-Bi_2_O_3_-5**). The latter is comparable to previously reported mesoporous β-Bi_2_O_3_ thin films (20–30 m^2^g^−1^) and thus is among the highest values reported for β-Bi_2_O_3_.[20] However, nitrogen adsorption measurements reveal type II isotherms, indicating the formation of nonporous β-Bi_2_O_3_ nanoparticles (see Figure S10, S11). The absorption edge [(542±2) nm; see Figure S12] and the optical band gaps are not significantly influenced by the crystallite size. The indirect band gap amounts to (2.15±0.05) eV and the direct band gap to (2.44±0.03) eV (see Table 1, see Figure S13, S14). A complete summary of the measured properties is given in Table 1. Notably, the calculation of the band gaps was carried out by Tauc plots,[21] in order to estimate the allowed indirect and direct band gap by plots of (*αhυ*)^1/2^ versus *hυ* and (*αhυ*)^2^ versus *hυ*, respectively. A plot of the absorption coefficient versus wavelength offers the possibility to investigate the behavior of the band gap of a semiconductor material.[22] A sharp onset of the absorption at the band gap energy (*E*_g_) combined with a large absorption coefficient for *hυ*> *E*_g_ is typical for a direct band gap semiconductor. An indirect band gap semiconductor shows a broad, weak onset of absorption which starts at *hυ*≤*E*_g_. A typical plot of the absorption coefficient versus wavelength and (*αhυ*)^1/2^ versus *hυ* is given for **β-Bi_2_O_3_-10** in Figure 3. This represents a typical indirect band gap semiconductor behavior similar to that recently proposed for β-Bi_2_O_3_ materials.[23] George et al. have reported on an indirect band gap of (1.74±0.05) eV for β-Bi_2_O_3_ thin films, which is significantly lower compared with that of the as-prepared β-Bi_2_O_3_ nanoparticles [(2.15±0.05) eV] and might be attributed to the different morphologies.[23a] The direct band gap values for the as-prepared samples [(2.44±0.03) eV] are comparable to the values for β-Bi_2_O_3_ nanowires (diameter≍7 nm) reported by Qiu et al. (2.47 eV), but significantly lower than the values for β-Bi_2_O_3_ films described by Brezesinski et al. (3.4 eV).[12e, 20]

**Table 1:** Crystallite size, BET surface area and photocatalytic performance of the as-prepared β-Bi_2_O_3_ samples

Sample	Time [min]^[a]^	Size [nm]^[b]^	BET surface area [m^2^ g^−1^]	*c*/*c*_0_ of RhB [%]^[c]^	*k*_1_ [s^−1^]
**β-Bi_2_O_3_-5**	5	17±2 (20±3)	29	0	4.01×10^−4^
**β-Bi_2_O_3_-10**	10	18±2 (21±3)	29	0	4.01×10^−4^
**β-Bi_2_O_3_-30**	30	23±2 (23±4)	26	1	4.00×10^−4^
**β-Bi_2_O_3_-120**	120	34±3 (33±4)	20	2	3.71×10^−4^
**β-Bi_2_O_3_-180**	180	40±4 (38±6)	10	14	2.07×10^−4^
**β-Bi_2_O_3_-240**	240	43±5 (n.m.)*	7	16	1.97×10^−4^
**β-Bi_2_O_3_-300**	300	45±5 (40±7)	7	19	1.78×10^−4^

[a] Annealing time at 370 °C. [b] Crystallite size determined by Scherrer’s equation (PXRD) and TEM (values in brackets). [c] After 150 min. [*] n.m.=not measured.

The photocatalytic activities of the as-prepared samples were investigated by UV/Vis studies on the degradation of a 10^−5^ m aqueous solution of rhodamine B (RhB) under visible light irradiation (420 nm≤*λ*≤700 nm). RhB is degraded by photooxidation processes that can follow two principle pathways.[24] A decrease of the intensity of the characteristic absorption band at 553 nm, which is accompanied by a sequential blueshift, implies a de-ethylation process of the *N,N,N′,N′*-tetraethylrhodamine. The degradation products, *N*,*N*,*N′*-triethylrhodamine (*λ*_max_=539 nm), *N*,*N*′-diethylrhodamine (*λ*_max_=522 nm), *N*-ethylrhodamine (*λ*_max_=510 nm), and rhodamine (*λ*_max_=498 nm), will induce a shift to lower wavelengths.[25] Another pathway is given by a full decomposition to give CO_2_ and water, which results in a decrease of the absorption band without a shift in wavelength.

**Figure 4:**
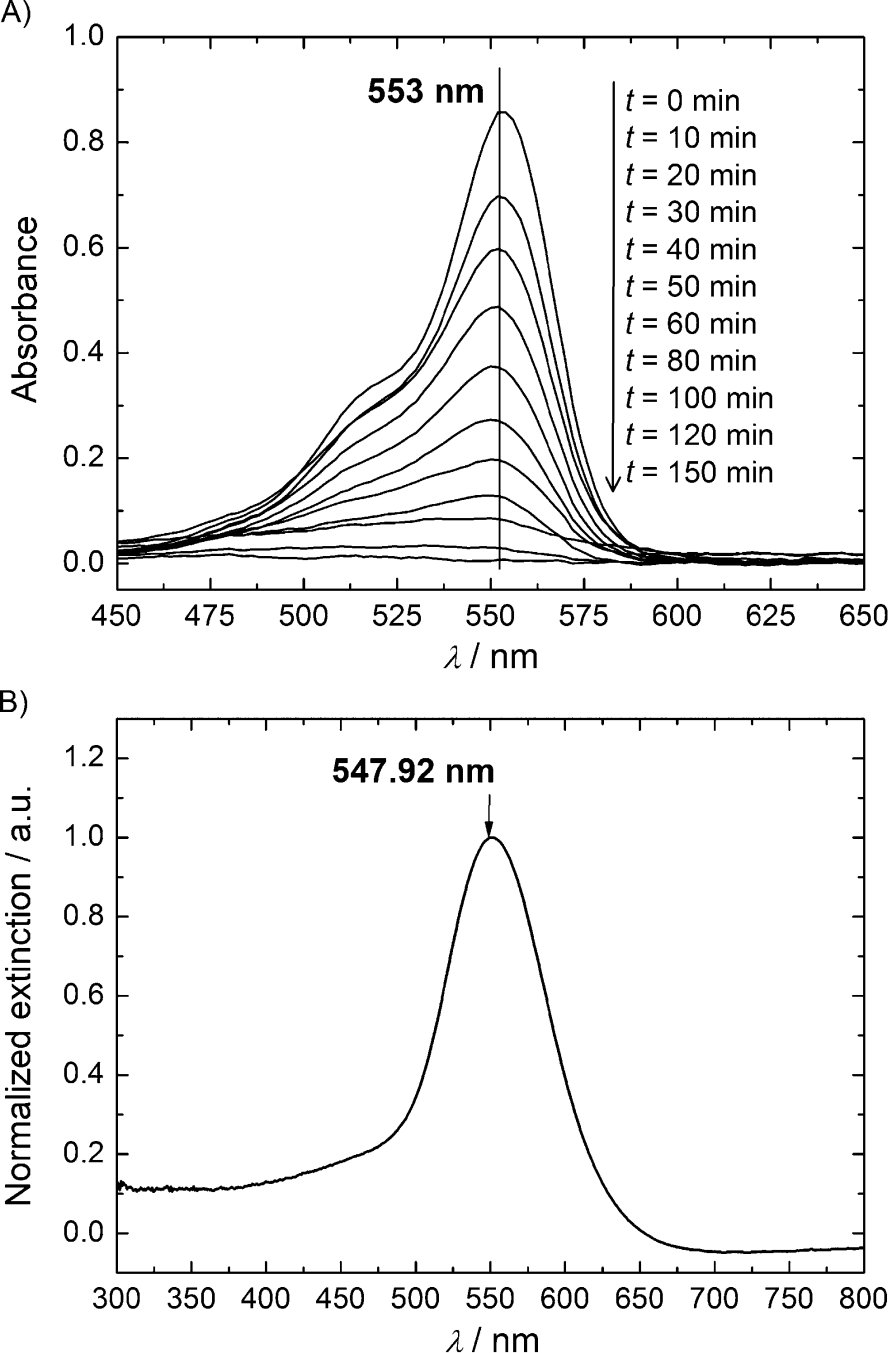
A) UV/Vis spectra of the photodegradation of RhB using sample **β-Bi_2_O_3_-10** as photocatalyst (*t*=−30 min: start of stirring in the dark; *t*=0 min: start of irradiation with visible light). B) UV/Vis absorption spectra of dicyanobis(1,10-phenanthroline)iron(II) complex adsorbed onto **β-Bi_2_O_3_-120**.

**Figure 5:**
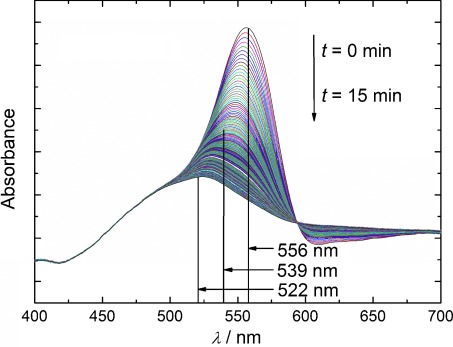
Diffuse reflectance UV/Vis spectra of solid **β-Bi_2_O_3_-10** with adsorbed RhB.

A decrease of the RhB absorption band at 553 nm without a blueshift is observed for all samples, exemplarily shown for **β-Bi_2_O_3_-10** in Figure 4 A. This indicates a fast decomposition of the conjugated chromophore system. However, a detailed understanding of the degradation processes of RhB at the surface of β-Bi_2_O_3_ is still lacking. Thus, we have performed UV/Vis measurements in diffuse reflectance mode with **β-Bi_2_O_3_-10**, which was covered by adsorbed RhB on the surface prior to the investigation. As shown in Figure 5, a shift from 556 nm to 522 nm is observed within 15 min by continuous irradiation with the instrument light source (≍100 W). The observed blueshift of the absorption band, which represents the formation of *N*,*N*,*N*′-triethylrhodamine and *N*,*N*′-diethylrhodamine, indicates an ongoing de-ethylation process of RhB at the β-Bi_2_O_3_ surface. In solution, the blueshift is not observed, which might be a result of adsorption/desorption kinetics. We assume that the degradation process of RhB at the surface of the β-Bi_2_O_3_ nanoparticles is faster than the desorption processes of de-ethylated RhB intermediates.

**Figure 6:**
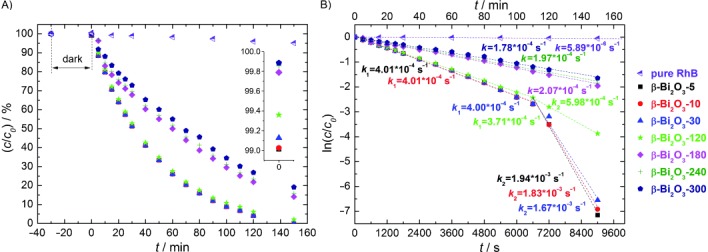
A) Time-dependent conversion and B) semilogarithmic plots of the photodegradation of an aqueous RhB solution (10^−5^ m) under visible light irradiation (*t*>0 min) using β-Bi_2_O_3_ nanoparticles with different crystallite sizes as photocatalysts. *t*≤0 min shows the adsorption behavior of the β-Bi_2_O_3_ nanoparticles for RhB (inset in A).

The normalized absorbance changes as a function of the irradiation time of the samples are given in Figure 6. The RhB solution was stirred for 30 min in the dark after addition of β-Bi_2_O_3_ nanoparticles to establish the adsorption/desorption equilibrium. The β-Bi_2_O_3_ samples do not show adsorption of a significant amount of RhB from the solution. However, studies in terms of a detailed characterization of β-Bi_2_O_3_ surfaces are still lacking in the literature. For Bi_2_WO_6_ and α-Bi_2_O_3_, a bismuth-rich surface with a high concentration of M–OH_ad_ (M=W, Bi) and H_2_O_ad_ is assumed.[26] With this assumption in mind, we probed the surface polarity for the as-prepared β-Bi_2_O_3_ nanoparticles. In general, the adsorption behavior of the dicyanobis(1,10-phenanthroline)iron(II) complex on a surface can be used to determine the hydrogen-bond-donating ability of a metal oxide surface, which is expressed as *α*. The value of *α* can be determined by using the Equation (1):[27]


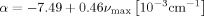
(1)

where *ν*_max_ represents the observed UV/Vis absorption maximum.

The adsorption of the dicyanobis(1,10-phenanthroline)iron(II) complex on the β-Bi_2_O_3_ nanoparticles results in a broad absorption band at 547.92 nm and thus gave *α*=0.91 (Figure 4 B). This value is significantly lower than that for silica gel 60 (*α*=1.14), Al_2_O_3_ (*α*=1.32), ZnO (*α*=1.56) and WO_3_ (*α*=1.62).[28] Thus, we assume that the hydrogen-bonding ability of the as-prepared β-Bi_2_O_3_ nanoparticles is lower compared to the other mentioned oxides. Note that, Saison et al. described the absence of Brønsted acid sites on α-Bi_2_O_3_ which might be taken as confirmation of our assumption that β-Bi_2_O_3_ nanoparticles show a low hydrogen-bonding ability.[6c]

The photocatalytic experiment under irradiation with visible light (*t*>0 min) without addition of a catalyst shows negligible decomposition of RhB after 150 min (5 %; see Figure 6). The best photocatalytic activities are observed for β-Bi_2_O_3_ nanoparticles with a crystallite size of 17–34 nm (**β-Bi_2_O_3_-5**, **β-Bi_2_O_3_-10**, **β-Bi_2_O_3_-30**, **β-Bi_2_O_3_-120**), which degrade approximately 100 % of the initial RhB within 150 min. The β-Bi_2_O_3_ nanoparticles with a crystallite size of approximately 40–50 nm (**β-Bi_2_O_3_-180**, **β-Bi_2_O_3_-240**, **β-Bi_2_O_3_-300**) decompose between 80 % and 85 % of the initial amount of RhB. Differences in activity between individual samples are expressed in terms of reaction rate constants. As shown in Figure 6, the degradation process of the aqueous RhB solutions in the presence of the photocatalysts follows pseudo-first-order reaction kinetics. In the absence of any catalyst, the degradation process is quite slow (*k*=5.89×10^−6^ s^−1^). The addition of β-Bi_2_O_3_ accelerates degradation at the beginning of the reaction by a factor of approximately 70 (*k*_1_=4.01×10^−4^ s^−1^; **β-Bi_2_O_3_-5**). The lowest rate constant is observed for **β-Bi_2_O_3_-300** (*k*=1.78×10^−4^ s^−1^). The samples **β-Bi_2_O_3_-240** and **β-Bi_2_O_3_-180** show slightly faster kinetics with *k* values of 1.97×10^−4^ s^−1^ and 2.07×10^−4^ s^−1^, respectively. It is noteworthy that the samples with a crystallite size up to approximately 34 nm display two distinct linear regimes with two different rate constants *k*. At the beginning of the degradation the rate constants are determined to 4.01×10^−4^ s^−1^ (**β-Bi_2_O_3_-5**, **β-Bi_2_O_3_-10**), 4.00×10^−4^ s^−1^ (**β-Bi_2_O_3_-30**) and 3.71×10^−4^ s^−1^ (**β-Bi_2_O_3_-120**). After 110 min the reaction is accelerated by a factor of 4.5. This phenomenon was described in the literature previously and most likely results from strong light absorption of an intensively colored RhB solution at low degradation rates.[20, 29]

As might be expected, our investigations show a strong influence on the activity in dependence of the crystallite size. A smaller crystallite size results in higher surface areas and thus provides more active catalyst sites, which is expressed in higher degradation rates. All of the β-Bi_2_O_3_ nanoparticles tested possess an excellent photocatalytic activity and are quite significantly more active than the β-Bi_2_O_3_ nanoflakes synthesized by Chen et al. (degradation of 55 % after 120 min.), which have been tested under similar conditions for the photocatalytic degradation of aqueous RhB solutions.[13d]

### Influence of catalyst amount and initial RhB concentration

**Figure 7:**
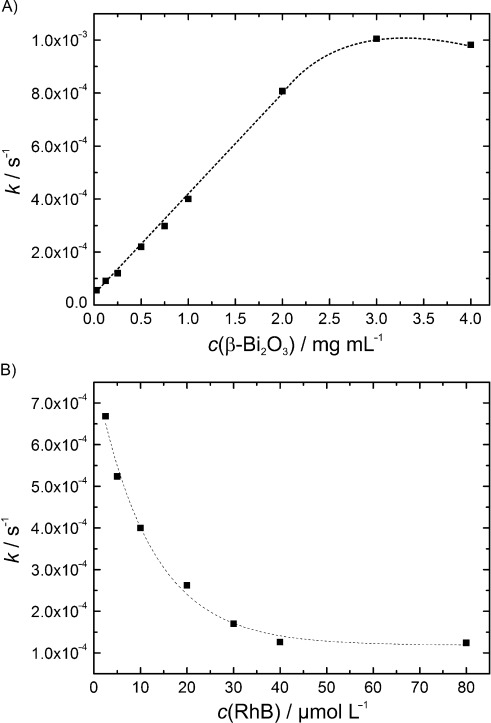
A) Plot of the rate constant versus the catalyst concentration (10^−5^ m RhB solution). B) Plot of the rate constant versus the initial RhB concentration (1.0 mg mL^−1^ **β-Bi_2_O_3_-10**).

The effect of varying the amount of the catalyst from 0.025 mg mL^−1^ to 4 mg mL^−1^ of β-Bi_2_O_3_ nanoparticles (**β-Bi_2_O_3_-10**) for the degradation of a 10^−5^ m aqueous solution of RhB was investigated. As shown in Figure 7 A, the efficiency of the degradation process increases with an increasing amount of the catalyst. The rate constants vary from 2.46×10^−5^ s^−1^ (0.025 mg mL^−1^) to 4.48×10^−4^ s^−1^ (3 mg mL^−1^). A linear region of *c*(β-Bi_2_O_3_) versus *k* is observed up to a concentration of 2 mg mL^−1^. At higher catalyst concentrations, the reaction rates become independent from the photocatalyst concentration as a result of agglomeration of catalyst particles, which reduces the number of catalytically active sites. Furthermore, stronger absorption and light-scattering effects are present, which reduce the ability of light to fully penetrate the solution.[30] As a consequence, less OH^.^ radicals are formed. A maximum of the reaction rate constant at a specific amount of the catalyst was also reported in the literature. For tungsten-doped TiO_2_ and for β-Bi_2_O_3_ photocatalysts this value was determined to be 8 mg mL^−1^ and 2 mg mL^−1^, respectively.[23b, 30] In our studies, the maximum of the reaction rate constant was observed for **β-Bi_2_O_3_-10** with approximately 3 mg mL^−1^. The higher concentration as compared to the results reported for β-Bi_2_O_3_ particles (41 nm) by Eberl and Kisch is assigned to the smaller crystallite size of **β-Bi_2_O_3_-10**.[23b]

The photocatalytic degradation properties of the β-Bi_2_O_3_ nanoparticles (**β-Bi_2_O_3_-10**) were also investigated in dependence of the initial RhB concentration. As shown in Figure 7 B, the reaction rate constants decrease by increasing the initial RhB concentration. This might be a result of the light absorption of higher-concentrated RhB solutions.[29] Furthermore, the photocatalytically active sites are blocked, which results in a reduction in the interaction of photons.[20, 30–31] By using the Langmuir–Hinshelwood kinetic model the reaction parameters were determined by Equation (2):[30, 32]


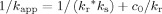
(2)

where *k*_app_ [s^−1^] is the apparent rate constant, *k*_r_ [μmol×L^−1^ s^−1^] is the reaction rate constant, *k*_s_ [L μmol^−1^] is the adsorption rate constant and *c*_0_ is the initial RhB concentration. A plot of *c*_0_ versus 1/*k*_app_ shows approximately linearity, which confirms the applicability of the Langmuir–Hinshelwood kinetic model for the investigated system (see Figure S15). By determining the intercept and the slope, the values of *k*_r_ and *k*_s_ were calculated to be 5.91×10^−3^ μmol×L^−1^ s^−1^ and 1.89×10^−1^ L μmol^−1^, respectively. For tungsten-doped TiO_2_, Li et al. observed values of 4.87×10^−3^ μmol L^−1^ s^−1^ and 6.23×10^−2^ L μmol^−1^ by using approximately eight-times higher catalyst concentrations as reported here.[30] However, the as-prepared β-Bi_2_O_3_ nanoparticles show a similar value for *k*_r_ and a significantly larger value for *k*_s_. This might express better adsorption properties along with higher degradation rates of β-Bi_2_O_3_ nanoparticles compared to tungsten-doped TiO_2_.

### Stability of β-Bi_2_O_3_ nanoparticles

**Figure 8:**
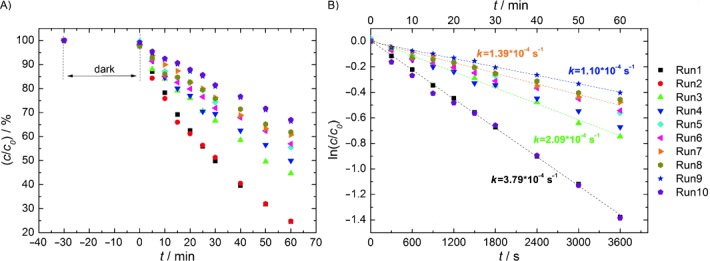
A) Time-dependent conversion and B) semilogarithmic plots of the photodegradation of an aqueous RhB solution (10^−5^ m) under visible light irradiation (*t*>0 min) using **β-Bi_2_O_3_-10** as photocatalyst in several runs. *t*<0 min shows the adsorption behavior of the **β-Bi_2_O_3_-10** nanoparticles towards RhB.

**Figure 9:**
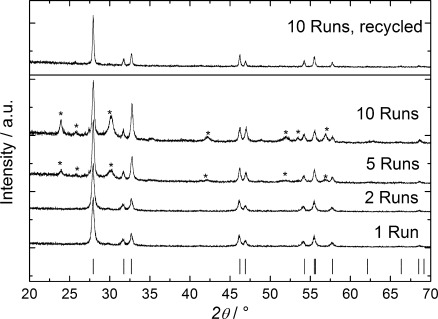
PXRD patterns of **β-Bi_2_O_3_-10** used in different numbers of catalytic cycles and recycled β-Bi_2_O_3_ after 10 runs (reference: β-Bi_2_O_3_, ICDD 00-027-0050). Asterisks represent reflections of Bi_2_O_2_CO_3_ (ICDD 00-041-1488).

The stability of the β-Bi_2_O_3_ nanoparticles in multiple photocatalytic cycles was tested by using 40 mg of the catalyst (**β-Bi_2_O_3_-10**) suspended in 40 mL of a 10^−5^ m aqueous RhB solution. After 60 min, the reaction is stopped and the catalyst isolated by centrifugation. The β-Bi_2_O_3_ particles are again dispersed in a 10^−5^ m RhB solution (40 mL) and exposed to visible light irradiation. As shown in Figure 8, the first two cycles exhibit identical photocatalytic performances with rate constants of 3.79×10^−4^ s^−1^. However, the following catalytic runs show a steady loss in photocatalytic activity. After ten catalytic cycles, only 33 % of the initial RhB is decomposed, resulting in a reaction rate constant of 1.10×10^−4^ s^−1^. One technical problem, but not the major one, is the partial loss of photocatalyst during the work-up procedure. After ten cycles, only 32 mg (80 %) of the catalyst was isolated. In a second experiment, a small amount of the photocatalyst after each cycle was used for PXRD studies. The diffraction patterns show the formation of Bi_2_O_2_CO_3_ after several catalytic cycles (Figure 9), which results in a step-by-step loss of the photocatalytic performance. For further consideration, Bi_2_O_2_CO_3_ nanoparticles were synthesized according to ref. [33] and tested in their photocatalytic degradation properties using our standard procedure. The Bi_2_O_2_CO_3_ nanoparticles only degrade approximately 45 % of a 10^−5^ m aqueous RhB solution within 60 min (see Figure S16), and thus a lower photocatalytic activity is obtained compared to **β-Bi_2_O_3_-10**. Based on the determination of the carbon content, it is assumed that the product consists of approximately 55 % β-Bi_2_O_3_ and 45 % Bi_2_O_2_CO_3_ after ten runs. It is suggested that the formation of Bi_2_O_2_CO_3_ results from the reaction of the β-Bi_2_O_3_ with the in situ formed CO_2_, which is released during the degradation process of RhB. For α-Bi_2_O_3_ and β-Bi_2_O_3_, this behavior was described recently by photodegradation of phenol after several catalytic cycles.[23b, 34] In the case of α-Bi_2_O_3_, formation of (BiO)_4_CO_3_(OH)_2_ and Bi_2_O_2_CO_3_ is described. β-Bi_2_O_3_ was reported to give α-Bi_2_O_3_, (BiO)_4_CO_3_(OH)_2_ and Bi_2_O_2_CO_3_. However, we observed Bi_2_O_2_CO_3_ as the only photocorrosion product in our experiments. Notably, Bi_2_O_2_CO_3_ shows a structural relationship to β-Bi_2_O_3_, and it was assumed that the catalyst might be easily recycled.[12a, 34a, 35] A temperature dependent PXRD study at a heating rate of 10 K min^−1^ of the Bi_2_O_2_CO_3_/β-Bi_2_O_3_ mixture shows the formation of phase-pure β-Bi_2_O_3_ between 370 °C and 380 °C. Above 420 °C, Bi_12_SiO_20_ is formed as a result of the reaction of the β-Bi_2_O_3_ nanoparticles with the quartz glass capillary (see Figure S17).[13e] Annealing of the Bi_2_O_2_CO_3_/β-Bi_2_O_3_ mixture at 380 °C for one hour in a furnace under argon atmosphere results quantitatively in phase-pure β-Bi_2_O_3_ with a crystallite size of (31±3) nm (Figure 9). The recycled β-Bi_2_O_3_ was tested in terms of the photocatalytic activity under the same conditions described above. As shown in Figure S18, the recycled β-Bi_2_O_3_ degrades approximately 67 % RhB within 60 min and gave a reaction rate constant of 3.16×10^−4^ s^−1^. The slightly slower degradation process compared to the starting β-Bi_2_O_3_ nanoparticles can be explained by the larger crystallite size of the recycled β-Bi_2_O_3_, but its photocatalytic activity is still quite high.

### Photocatalytic degradation of selected organic pollutants

**Figure 10:**
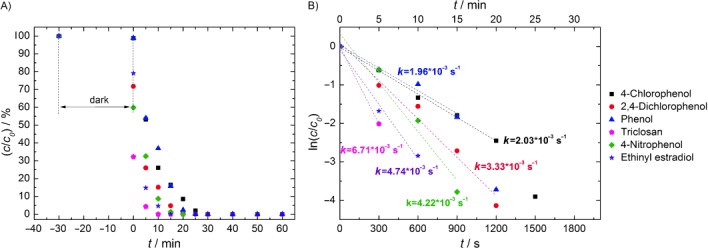
A) Time-dependent conversion and B) semilogarithmic plots of the photodegradation of phenol, 4-chlorophenol, 2,4-dichlorophenol, 4-nitrophenol, triclosan and ethinyl estradiol under visible light irradiation (*t*>0 min) using β-Bi_2_O_3_ nanoparticles as photocatalyst.

A photocatalyst with potential applications in the field of water treatment has to be active in the degradation of different organic substances. Our results demonstrate that β-Bi_2_O_3_ nanoparticles show a high photocatalytic activity in the degradation of 4×10^−5^ m aqueous solutions of various model dyes such as orange G, methylene blue, methyl orange and rhodamine B.[13e] Thus, we report on the extension of our investigations to typical organic water pollutants such as phenol, 4-chlorophenol, 2,4-dichlorphenol, 4-nitrophenol, triclosan, and ethinyl estradiol. These pollutants were demonstrated to be present in water and are acting as endocrine disruptors, which for example results in feminization of male fish.[18, 36] The photocatalytic degradation experiments were performed using 40 mL of an 4×10^−5^ m aqueous solution of the appropriate pollutant and 40 mg of β-Bi_2_O_3_ nanoparticles (**β-Bi_2_O_3_-10**) as catalyst. The organic pollutants are completely decomposed within 30 min (Figure 10). The highest rate constant is observed for the decomposition of triclosan (*k*=6.71×10^−3^ s^−1^) followed by that of ethinyl estradiol (*k*=4.74×10^−3^ s^−1^), 4-nitrophenol (*k*=4.22×10^−3^ s^−1^) and 2,4-dichlorophenol (*k*=3.33×10^−3^ s^−1^). The high activity results from the effective adsorption of the pollutants at the surface of the β-Bi_2_O_3_ nanoparticles and might be explained by bismuth π interactions of the present aromatic system with a bismuth-rich surface.[37] The lowest rate constants are observed for 4-chlorophenol (*k*=2.03×10^−3^ s^−1^) and phenol (*k*=1.96×10^−3^ s^−1^). β-Bi_2_O_3_ particles prepared by Cheng et al. are reported to decompose approximately 80 % of a 1.56×10^−4^ m aqueous solution of 4-chlorophenol within 90 min under similar conditions.[13a] Eberl and Kisch described a 94 % mineralization of a 3.13×10^−4^ m aqueous solution of 4-chlorophenol within 2 h by irradiation at a wavelength of *λ*≥455 nm.[23b] Li et al. investigated BiOI/Bi_2_O_3_ heterostructures in terms of their photocatalytic behavior in the decomposition of phenol and 4-chlorophenol under visible light irradiation using a 500 W xenon lamp.[38] The BiOI/Bi_2_O_3_ sample containing 20 % BiOI, which exhibits the best photocatalytic activity, was reported to give reaction rate constants of 8.4×10^−5^ s^−1^ and 2.6×10^−5^ s^−1^ in the degradation of phenol and 4-chlorophenol, respectively. However, the meaningful comparison of obtained rate constants with reported values is barely possible as a result of the lack of standardized reactor systems and procedures.

## Conclusions

Detailed information about the degradation parameters and the decomposition of typical organic pollutants using β-Bi_2_O_3_ particles are scarce. Here, we have shown the synthesis of β-Bi_2_O_3_ nanoparticles starting from the well-defined bismuth oxido cluster [Bi_38_O_45_(OMc)_24_(DMSO)_9_]⋅2 DMSO⋅7 H_2_O[16] with control of the crystallite size between (17 nm±2) nm and (45±5) nm and BET surface areas between 29 m^2^g^−1^ and 7 m^2^g^−1^. The indirect band gap was determined to (2.15±0.05) eV. The β-Bi_2_O_3_ nanoparticles were used as photocatalysts to investigate the influence of the crystallite size, the concentration of the catalyst and the concentration of the dye solution using rhodamine B (RhB) dye as a model system. A decrease of the crystallite size, a higher concentration of the catalyst as well as lower concentrations of RhB solutions result in significantly higher decomposition rates. Especially at high concentrations of RhB solutions, absorption of light limits the photocatalytic activity. β-Bi_2_O_3_ is transformed into Bi_2_O_2_CO_3_ after several catalytic cycles, which resulted in lower decomposition rates. However, the photocatalyst can be easily recycled by annealing under argon atmosphere at 380 °C for one hour. The recycled β-Bi_2_O_3_ (crystallite size (31±3) nm) shows only marginally lower performance compared with as-prepared β-Bi_2_O_3_. The β-Bi_2_O_3_ nanoparticles photocatalytically decompose phenol, 4-chlorophenol, 2,4-dichlorphenol, 4-nitrophenol, triclosan and ethinyl estradiol with excellent degradation rates. The photooxidation properties, the possibility to recycle the catalyst as well as the opportunity of a straightforward gram scale production demonstrate that the here presented β-Bi_2_O_3_ nanoparticles are auspicious materials for water purification photocatalyst systems. Preliminary investigations by irradiation with sun light over a period of seven hours showed promising activities in the degradation of RhB (see Figure S19). Further studies are currently under progress to develop an efficient photocatalytic system on the basis of immobilized β-Bi_2_O_3_ nanoparticles to reduce the leaching as observed upon multiple catalytic cycles.

## Experimental Section

**General**: Powder X-ray diffraction (PXRD) patterns were measured with a STOE Stadi P diffractometer (Darmstadt, Germany) using Cu K_*α*_ radiation (40 kV, 40 mA) and a Ge(111)-monochromator. The crystallite size was estimated using the formula determined by the Scherrer equation 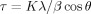, where *τ* is the volume-weighted crystallite size [nm], *K* is the Scherrer constant, here taken as 1.0, *λ* is the X-ray wavelength, *θ* is the Bragg angle and *β* is the full width of diffraction line at half of the maximum intensity (FWHM; background subtracted). The FWHM is corrected for instrumental broadening using a LaB_6_ standard (SRM 660) purchased from the US National Institute of Standards and Technology (NIST). The value of *β* was corrected from 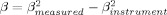 (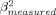 and 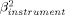 are the FWHMs of measured and standard profiles). Transmission electron micrograms were obtained by a 200 kV high-resolution transmission electron microscope (HRTEM; CM 20 FEG, Philips) with an imaging energy filter from Gatan (GIF, CA, USA). The energy dispersive X-ray (EDX) spectroscopy experiments and morphology investigations were examined using a scanning electron microscope (SEM; NanoNovaSEM, FEI, OR, USA). Specific surface analyses were performed at liquid nitrogen temperature (77 K) using a Micromeritics Gemini 2370 (GA, USA), which were evaluated by the Brunauer–Emmett–Teller (BET) method in the *p*/*p*_0_ range of 0.001–0.25. The adsorption/desorption isotherms were recorded at liquid nitrogen temperature (−196 °C) after activation under vacuum at 130 °C for 1 h using a Sorptomatic 1990 (Fisons Instruments, Ipswich, UK). Diffuse reflectance UV/Vis spectroscopy was performed using a single-beam simultaneous spectrometer MCS 400 (Carl Zeiss Jena GmbH). The UV and Vis radiation were generated using a deuterium lamp CLD 300 and a xenon lamp CLX 11, respectively. CHN analyses were obtained with a Thermo Flash EA 1112 CHN analyzer (Thermo Fisher Scientific). The in situ UV/Vis measurements to examine the photocatalytic activity were carried out by using an Agilent Cary 60 UV/Vis (Agilent Technologies) equipped with fiber optics.

**Synthesis of β-Bi_2_O_3_ nanoparticles**: The precursor [Bi_38_O_45_(OMc)_24_ (DMSO)_9_]⋅2 DMSO⋅7 H_2_O was synthesized according to the literature.[16] In a typical procedure, the precursor was converted into β-Bi_2_O_3_ nanoparticles as published previously.[13e] In order to control the particle size, the time of temperature annealing at 370 °C was varied between 5 min and 5 h. PXRD analyses proved the formation of phase-pure β-Bi_2_O_3_ in every case. CHN and EDX analyses revealed that the as-prepared products are free of carbon and sodium, respectively.

**Photocatalytic tests**: The photodegradation experiments were carried out by using 40 mL of an aqueous solution of 1×10^−5^ m rhodamine B (RhB) or 4×10^−5^ m aqueous solutions of the appropriate organic pollutant and 40 mg of the as-prepared samples in a water-cooled glass reactor (15 °C). If not further specified, β-Bi_2_O_3_ nanoparticles with a crystallite size of approximately 20 nm were used. The suspension was stirred in the dark for 30 min to reach the adsorption/desorption equilibrium. The suspension was illuminated with a 300 W xenon lamp (Cermax® VQTM ME300BF, PerkinElmer) equipped with a hot mirror filter (*λ*≤700 nm) and a UV cutoff filter (*λ*≥420 nm, GG420, Schott) to provide visible light irradiation. The effective irradiation area was 4.52 cm^2^ (≍25 % of the reactor area). The UV/Vis measurements were carried out in situ by stopping to stir for 10 s and darkening the light beam using a cover. The measurements were carried out up to 150 min. Up to 30 min measurements were done with a 5 min interval and up to 120 min with a 10 min interval. The degrees of conversion were determined by calculating the mathematical area under the characteristic UV/Vis absorption bands of the appropriate compounds.

## Supplementary material

As a service to our authors and readers, this journal provides supporting information supplied by the authors. Such materials are peer reviewed and may be re-organized for online delivery, but are not copy-edited or typeset. Technical support issues arising from supporting information (other than missing files) should be addressed to the authors.
