# Supplementary material for: Metastable β-Bi2O3 Nanoparticles with Potential for Photocatalytic Water Purification Using Visible Light Irradiation
Source: ChemistryOpen. 2013 Jul 2;2(4):146–55. doi: 10.1002/open.201300013 (PMC3775521; doi:10.1002/open.201300013)
Supplement: Supplementary file 1 [file open0002-0146-SD1.pdf]

Supporting Information

© 2013 The Authors. Published by Wiley-VCH Verlag GmbH & Co. KGaA, Weinheim

Metastable β-Bi_2O_3 Nanoparticles with Potential for Photocatalytic Water Purification Using Visible Light Irradiation

Maik Schlesinger,[a] Marcus Weber,[a] Steffen Schulze,[b] Michael Hietschold,[b] and Michael Mehring*[a]

open_201300013_sm_miscellaneous_information.pdf

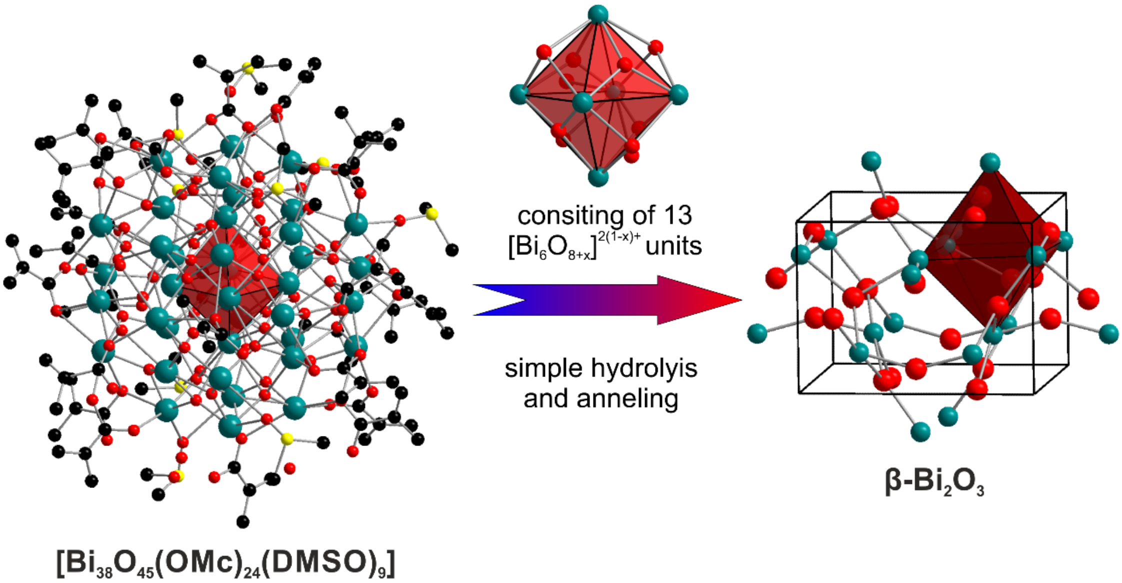

Figure S1. Structural relationship between $[Bi_{38}O_{45}(OMc)_{24}(DMSO)_9]·2DMSO·7H_2O$ and β-Bi_2O_3. The bismuth oxido cluster as well as β-Bi_2O_3 are composed of different numbers of the highlighted $[Bi_6O_{8-x}]^{2(1+x)+}$ motif. Extension of this structural motif to an infinite network based on such edge-sharing $[Bi_6O_{8-x}]^{2(1+x)+}$ units results in a nearly face centered cubic packing of the bismuth atoms similar to the structure of β-Bi_2O_3.

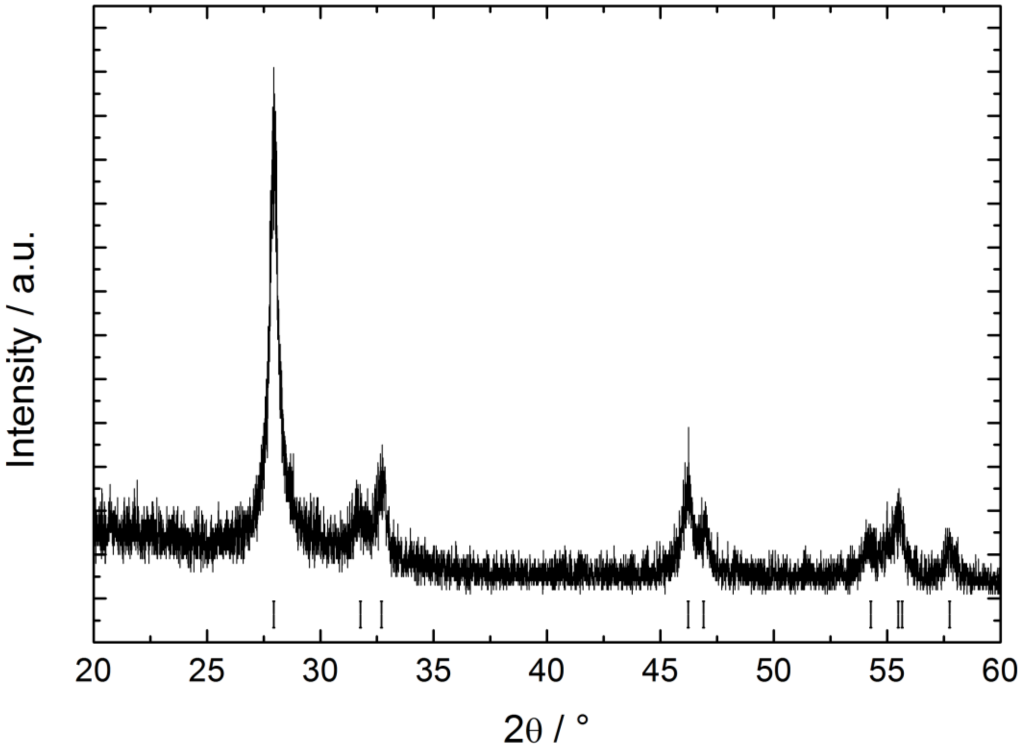

Figure S2. PXRD pattern of **β-Bi_2O_3-5** (reference: β-Bi_2O_3, ICDD 00-027-0050).

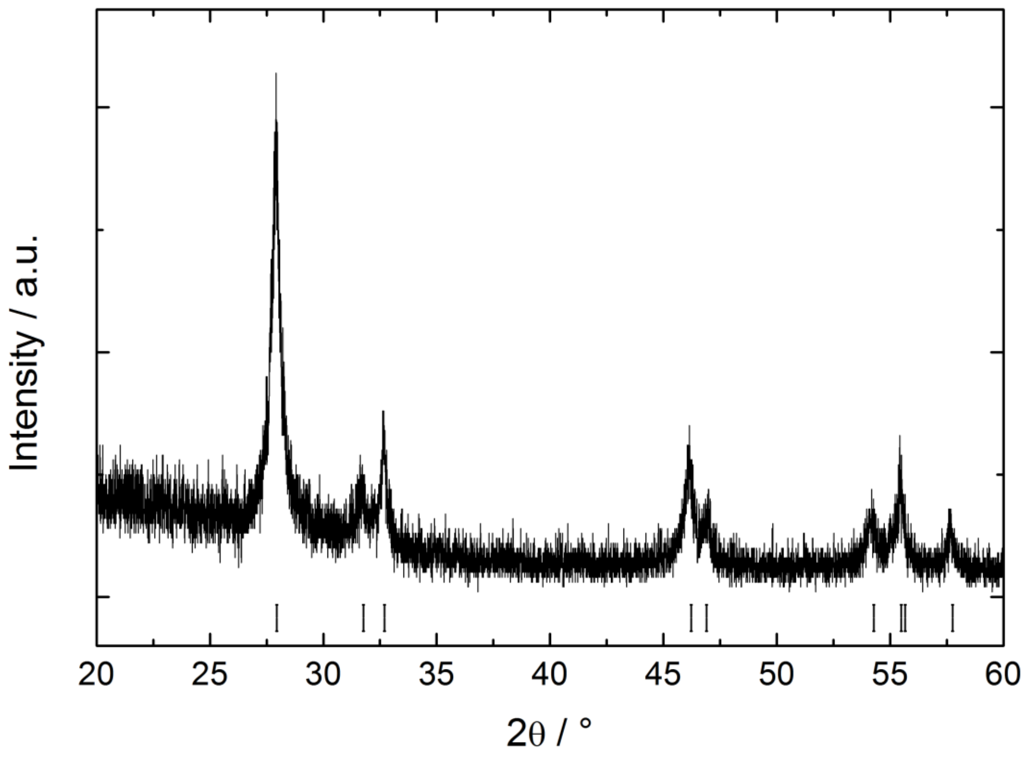

Figure S3. PXRD pattern of **β-Bi$_2$O$_3$-10** (reference: β-Bi$_2$O$_3$, ICDD 00-027-0050).

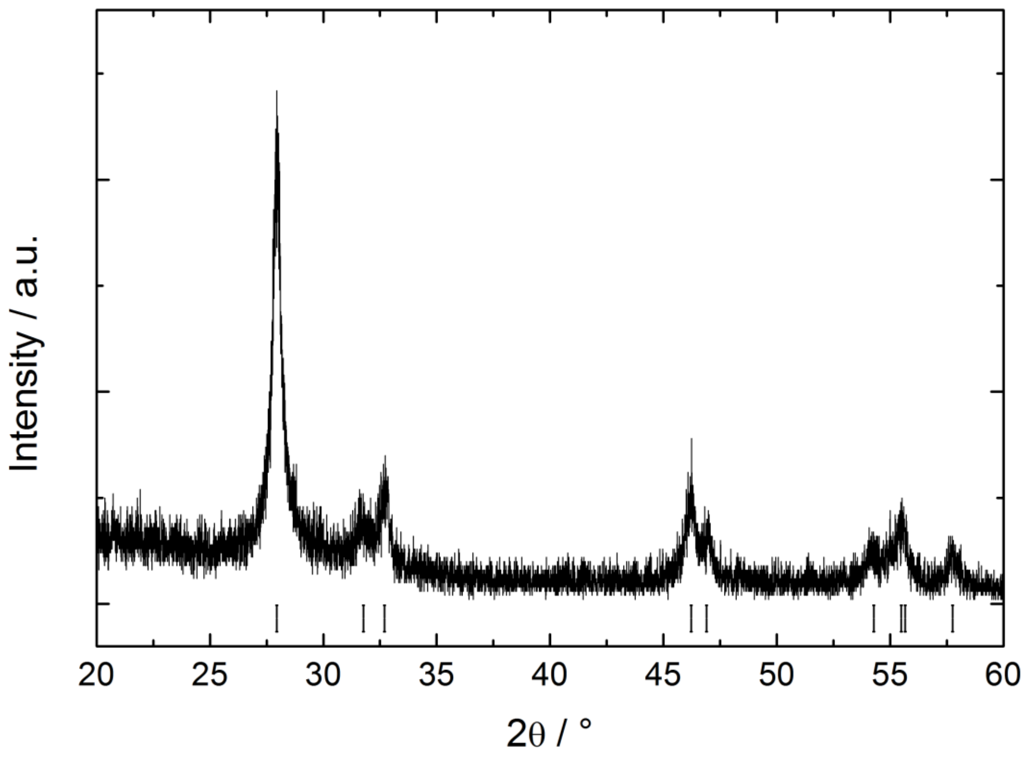

Figure S4. PXRD pattern of **β-Bi$_2$O$_3$-30** (reference: β-Bi$_2$O$_3$, ICDD 00-027-0050).

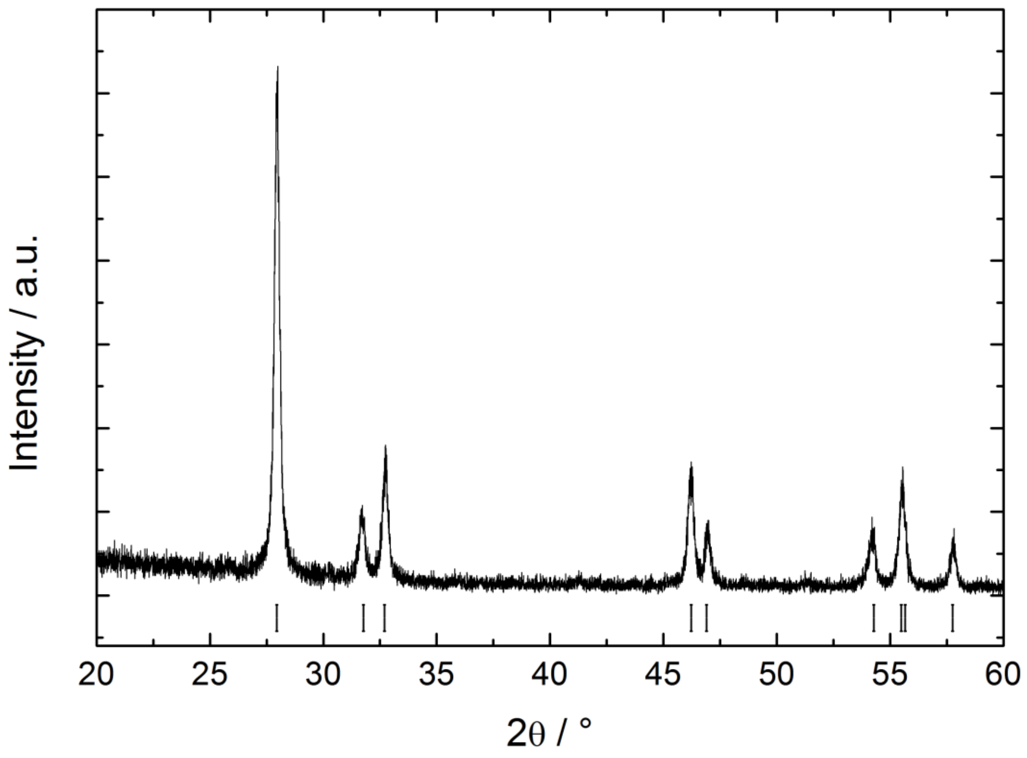

Figure S5. PXRD pattern of **β-Bi_2O_3-120** (reference: β-Bi_2O_3, ICDD 00-027-0050).

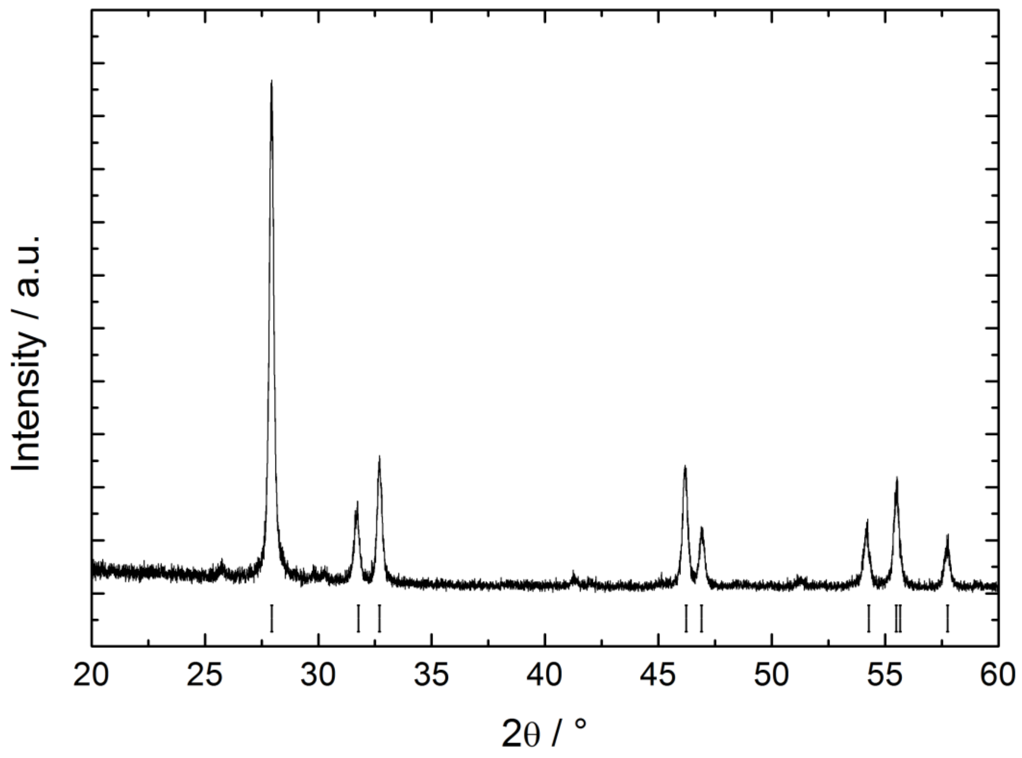

Figure S6. PXRD pattern of **β-Bi_2O_3-180** (reference: β-Bi_2O_3, ICDD 00-027-0050).

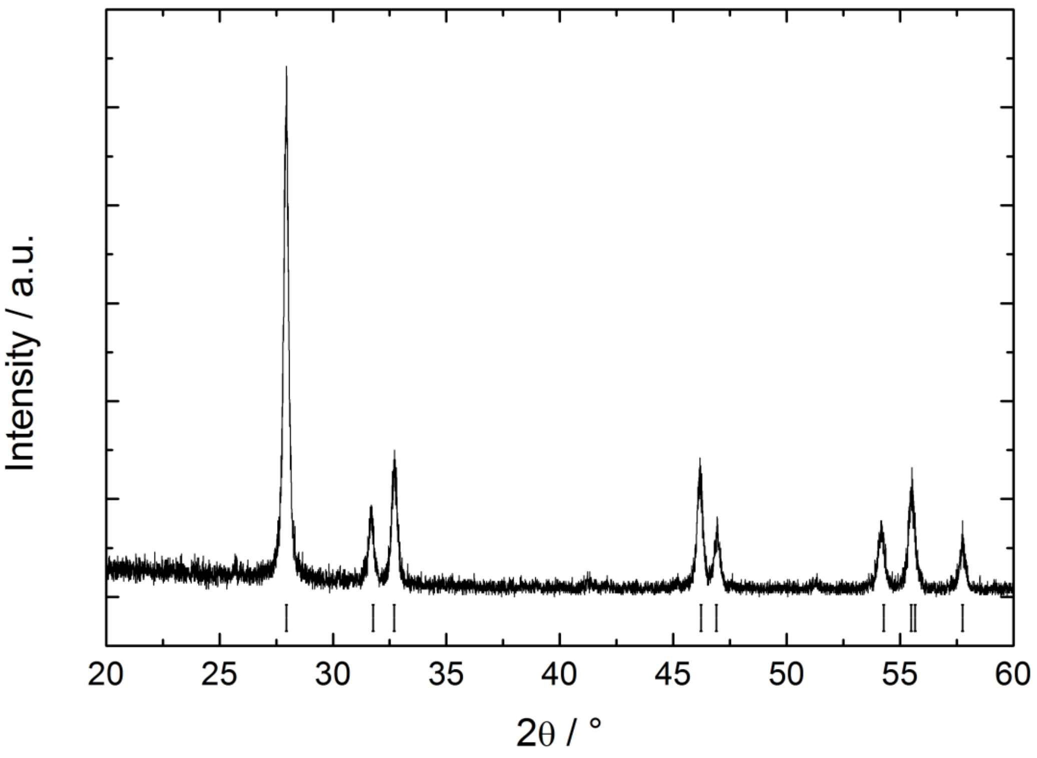

Figure S7. PXRD pattern of **β-Bi_2O_3-240** (reference: β-Bi_2O_3, ICDD 00-027-0050).

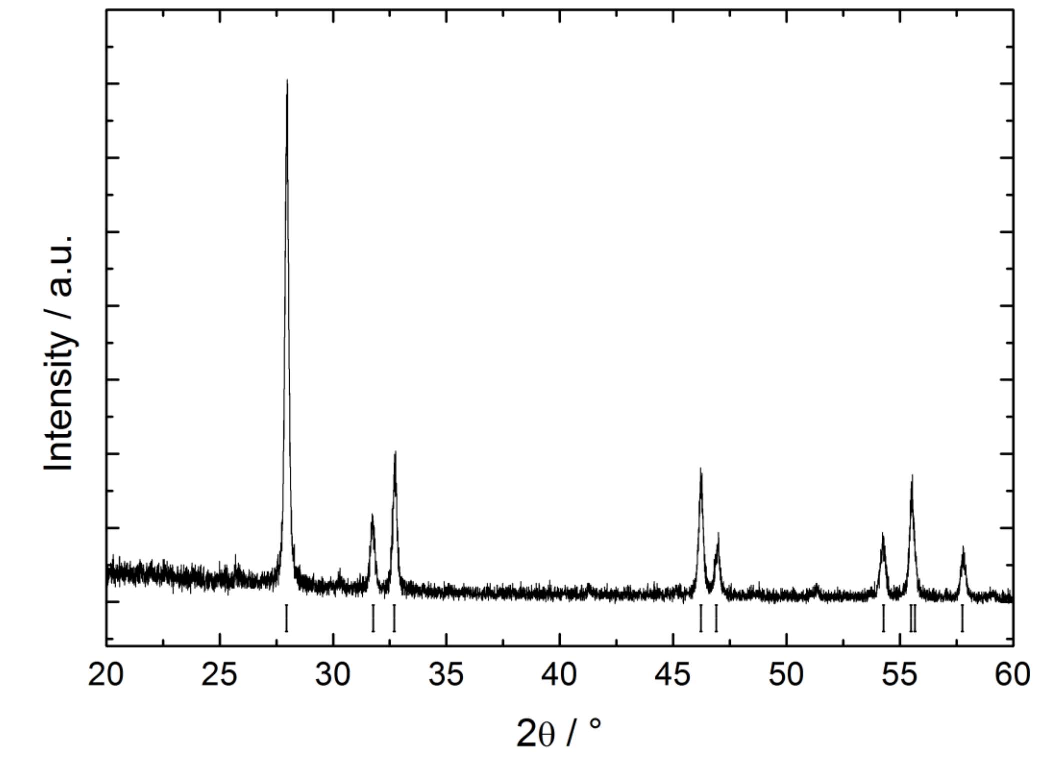

Figure S8. PXRD pattern of **β-Bi_2O_3-300** (reference: β-Bi_2O_3, ICDD 00-027-0050).

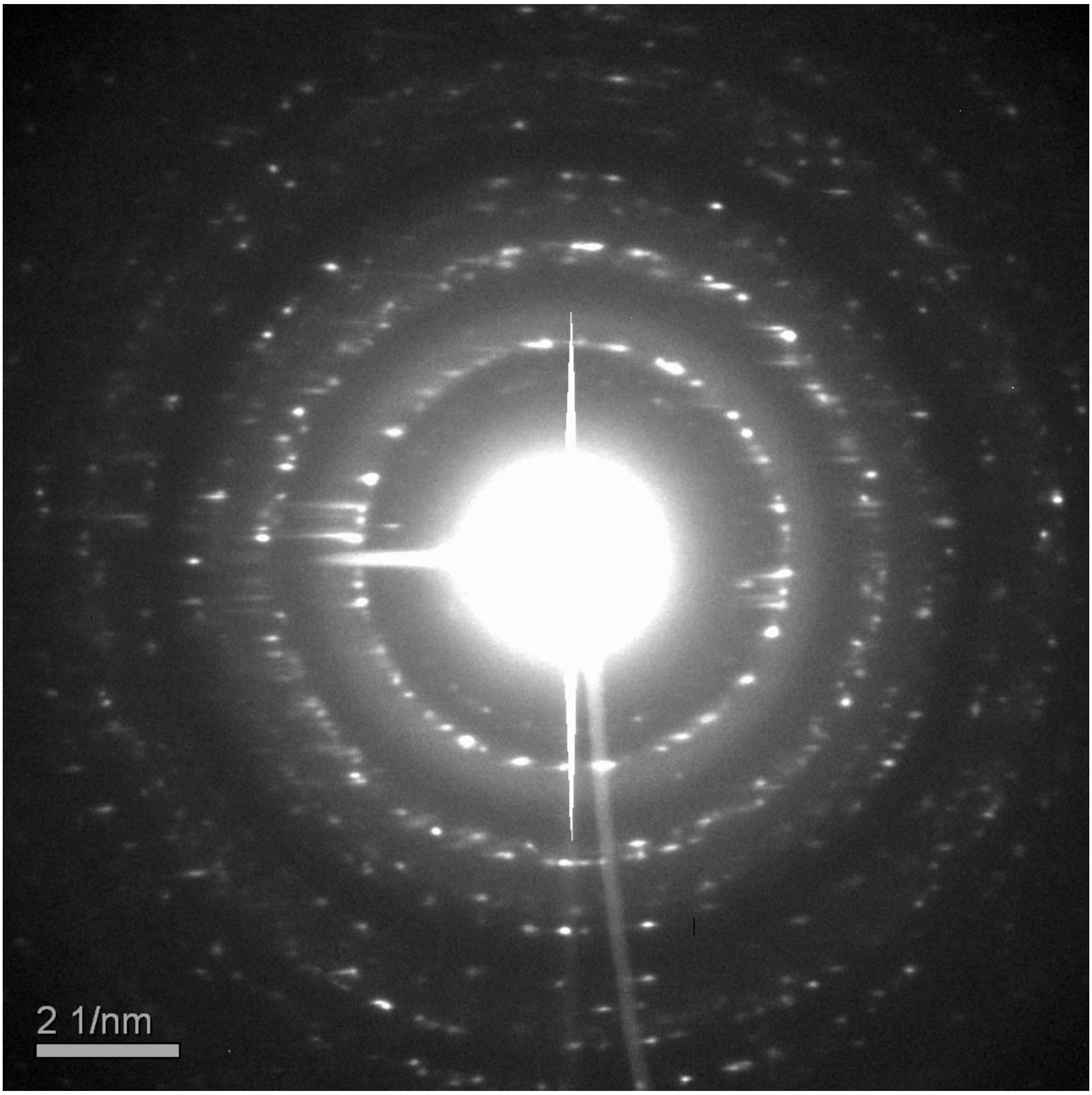

Figure S9. Electron diffraction image of sample **β-Bi_2O_3-5** that confirms the formation of the β-Bi_2O_3 polymorph.

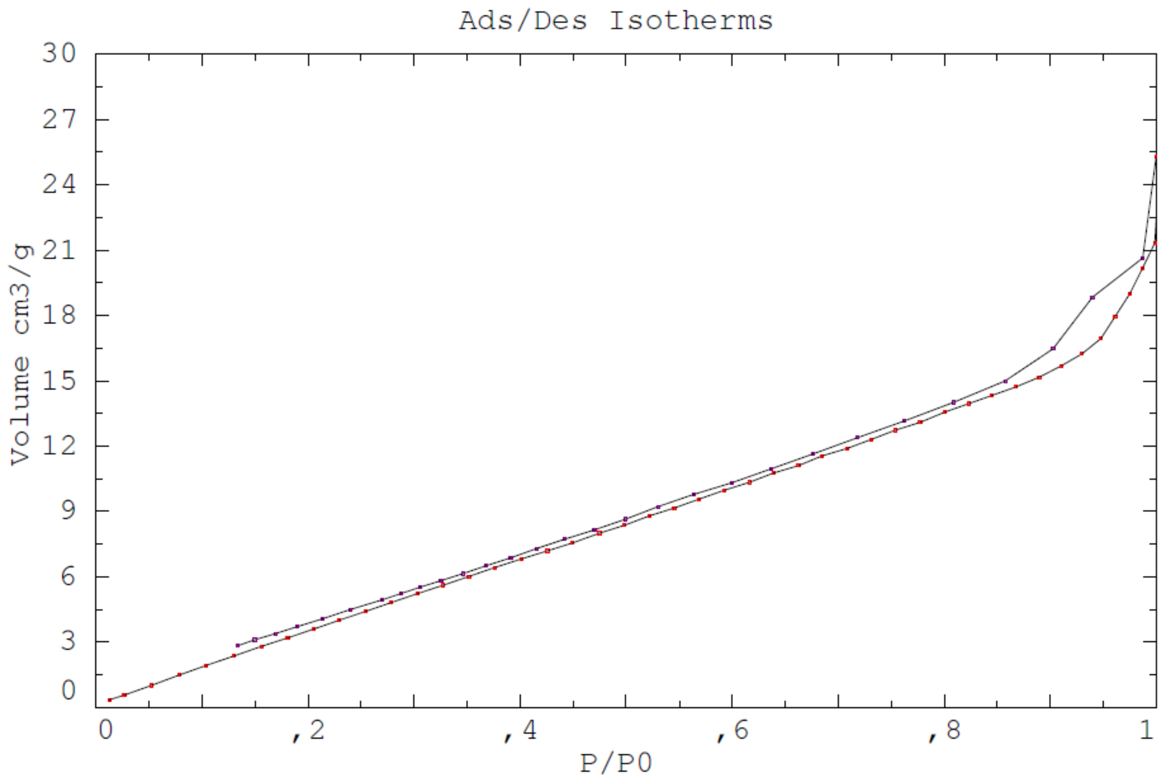

Figure S10. Nitrogen adsorption / desorption isotherm of sample **β-Bi_2O_3-5**.

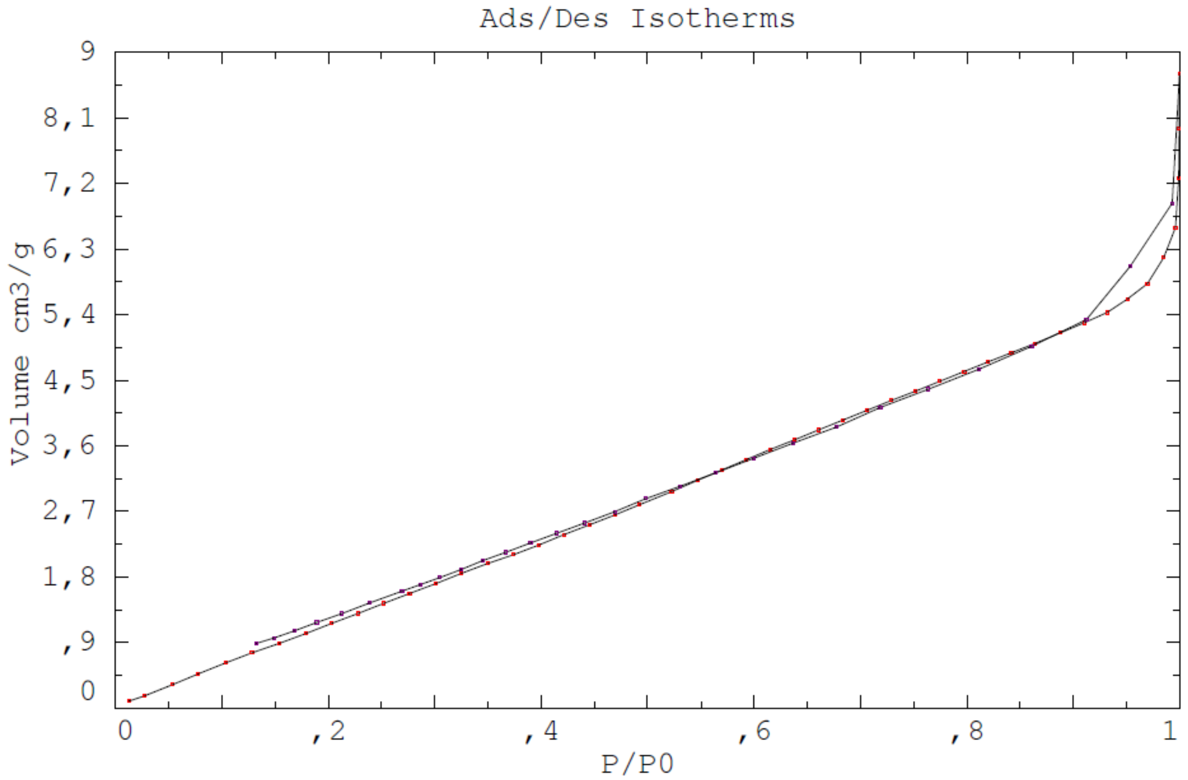

Figure S11. Nitrogen adsorption / desorption isotherm of sample **β-Bi_2O_3-300**.

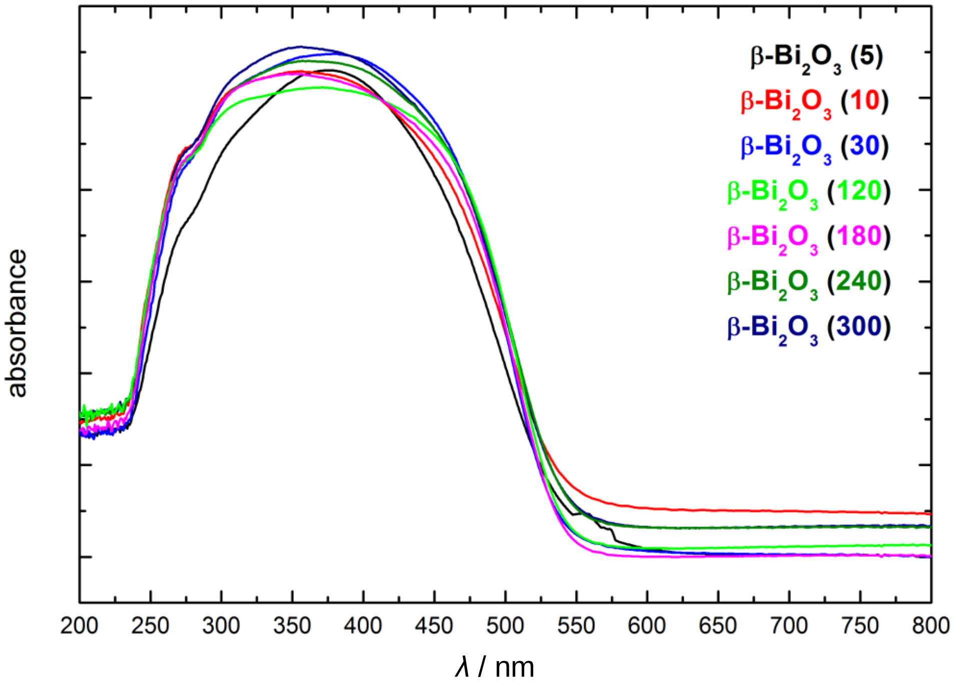

Figure S12. UV-Vis diffuse reflectance spectra of all investigated samples.

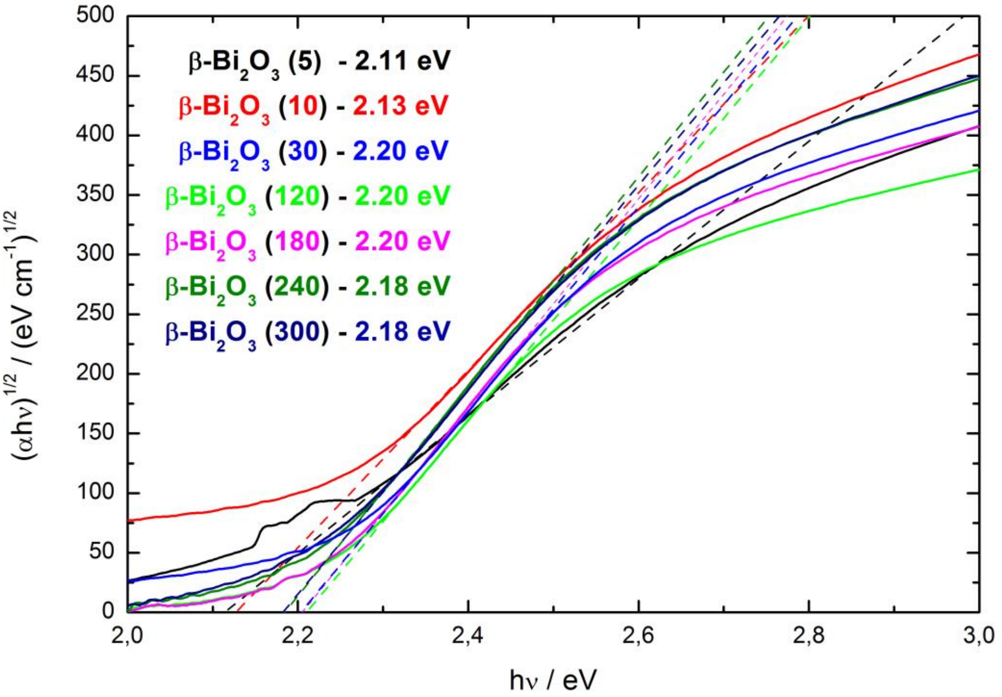

Figure S13. A plot of $(\alpha h\upsilon)^{1/2}$ vs. hυ (right) for all investigated samples (indirect band gap).

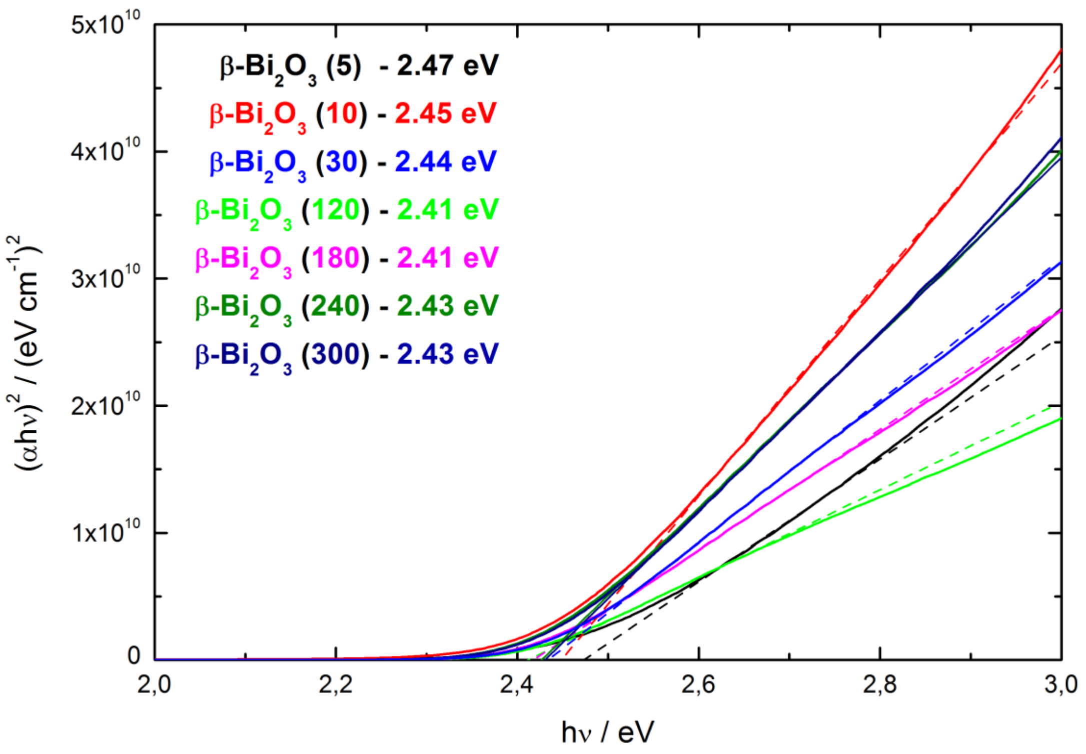

Figure S14. A plot of $(\alpha h\upsilon)^2$ vs. hυ (right) for all investigated samples (direct band gap).

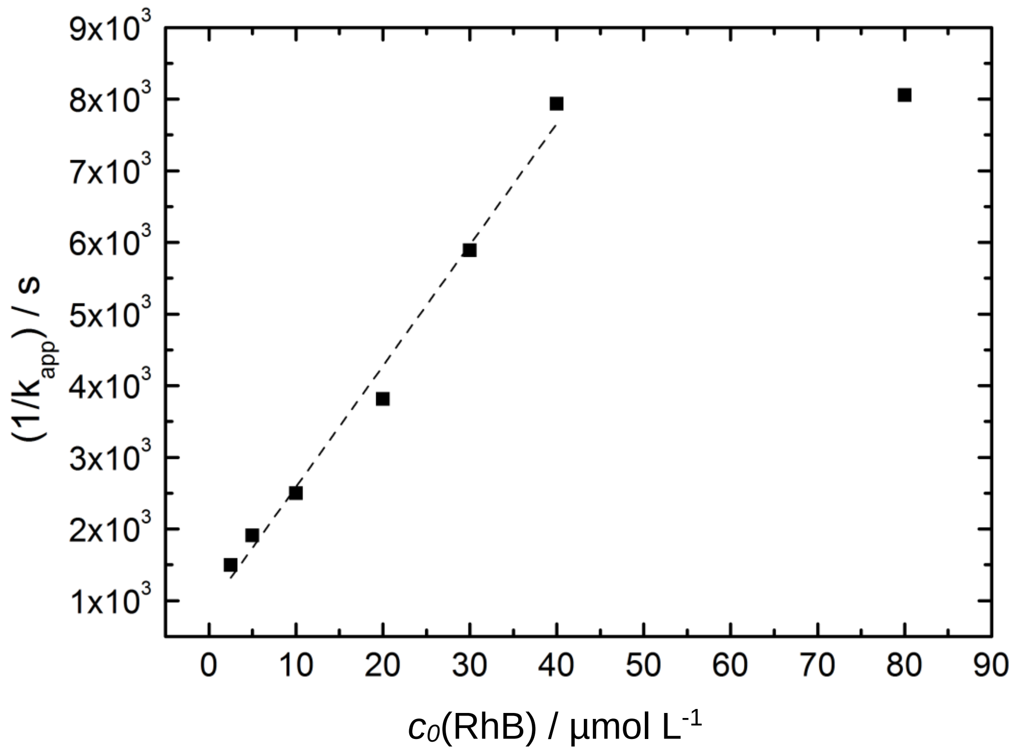

Figure S15. Reciprocal of the apparent rate constant vs. initial RhB concentration.

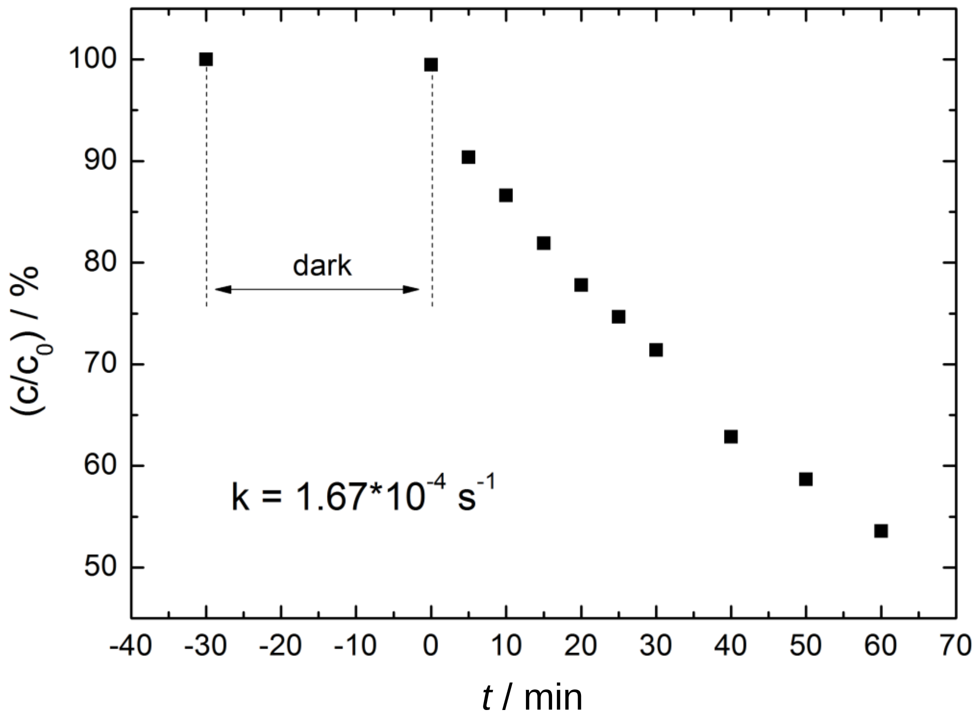

Figure S16. Time dependent conversion of the photodegradation of an aqueous RhB solution (10^{-5} M) under visible light irradiation (t > 0 min) using $Bi_2O_2CO_3$ nanoparticles as photocatalyst in several runs. t < 0 min shows the adsorption behavior of the $Bi_2O_2CO_3$ nanoparticles towards RhB.

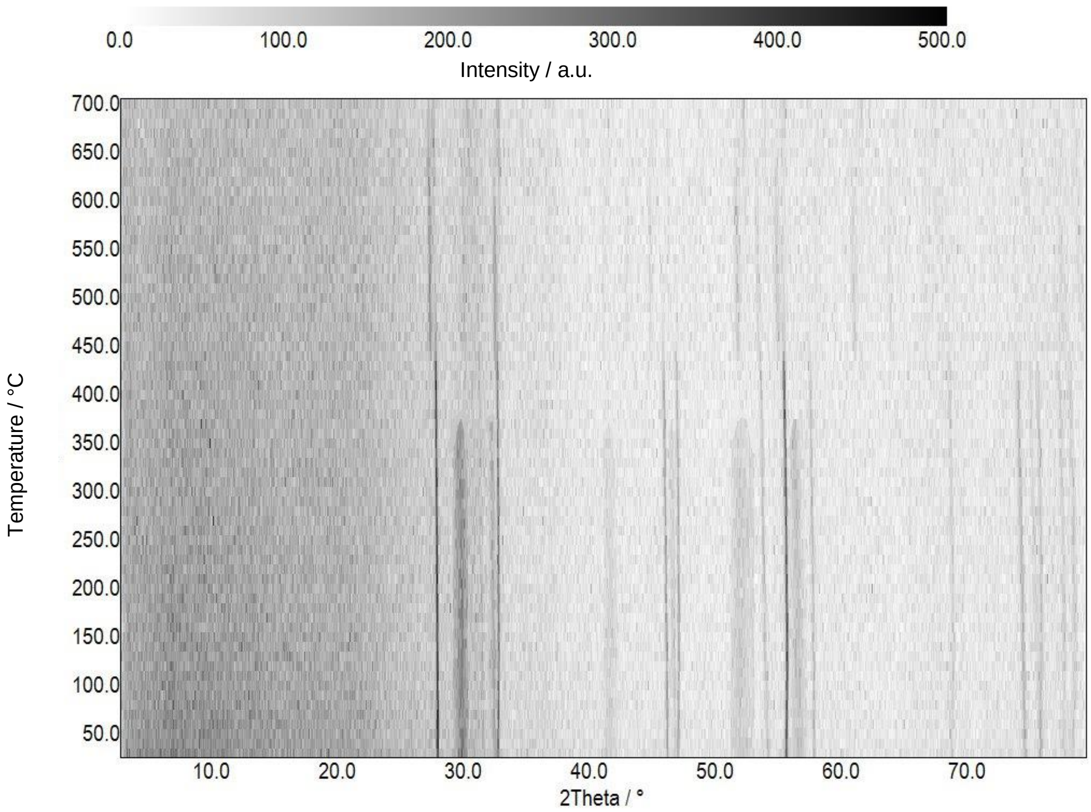

Figure S17. Temperature dependent powder X-ray diffraction experiment at **β-Bi_2O_3-10** which was used in ten photocatalytic cycles.

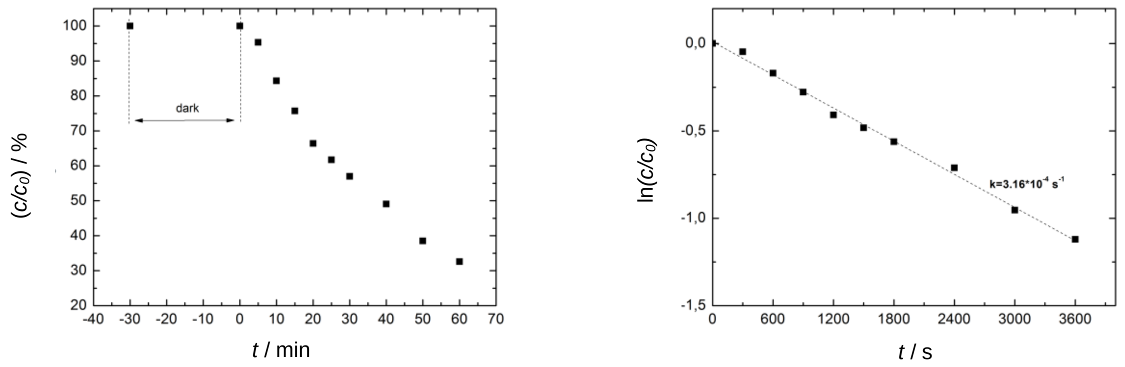

Figure S18. Time dependent conversion (*left*) and semilogarithmic plots (*right*) of the photodegradation of an aqueous RhB solution (10^{-5} M) under visible light (t > 0 min) using recycled β-Bi_2O_3 as photocatalyst. t < 0 min shows the adsorption behavior of the β-Bi_2O_3 nanoparticles towards RhB.

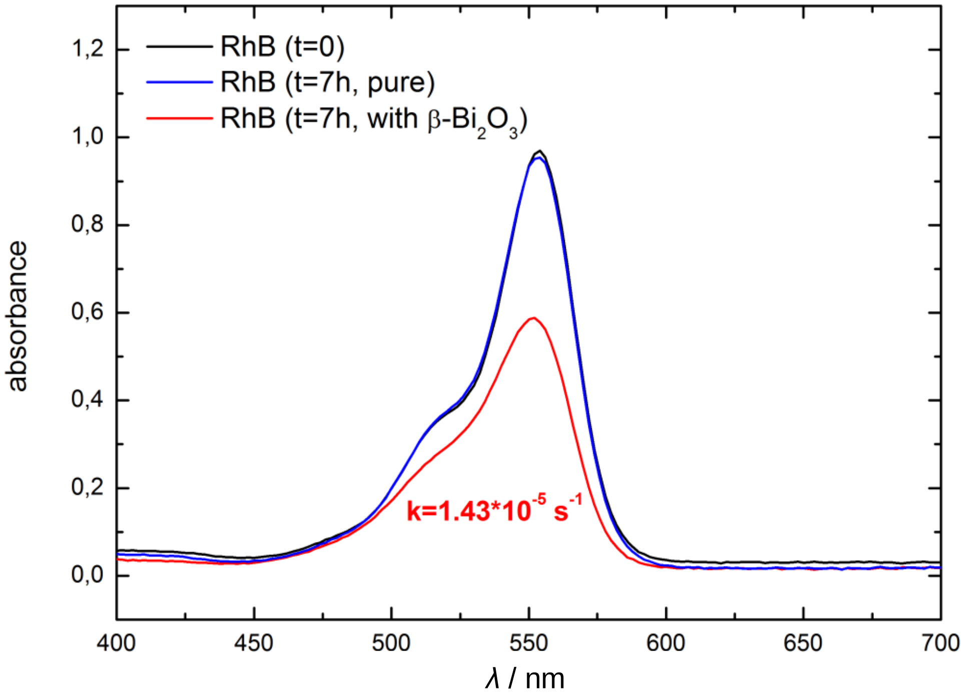

Figure S19. Photocatalytic degradation of RhB (10^{-5} M, 10 mL) without and with addition of 5 mg **β-Bi_2O_3-10** using sun light (period: 7h).
